# Impact of emotional divorce on the mental health of married women in Saudi Arabia

**DOI:** 10.1371/journal.pone.0293285

**Published:** 2023-11-10

**Authors:** Hend Faye AL-shahrani, Mohammad Ahmed Hammad

**Affiliations:** 1 Social Planning Department, Faculty of Social Services, Princess Nourah Bint Abdulrahman University, Riyadh, Saudi Arabia; 2 Mohammad Ahmed Hammad, Faculty of Education, Najran University, Najran, Saudi Arabia; 3 Assiut University, Assiut Governorate, Egypt; National University of Sciences and Technology, PAKISTAN

## Abstract

Emotional divorce occurs when physical, psychological, mental, and spiritual separation occurs between spouses despite the fact that they live in the same house and exercise their marital duties. Emotional divorce has adverse effects on the mental health of those involved, as evidenced by the higher incidence of depression, anxiety, and loneliness among such couples. Saudi women are particularly vulnerable to emotional divorce owing to social, legal, economic, and cultural factors. Therefore, it is important to examine the relationship between emotional divorce and mental health issues (depression, anxiety, and loneliness) in married women in Saudi Arabia. Using scales that assess emotional divorce, depression, anxiety, and loneliness, data were collected from 241 married Saudi women (M_age_ = 34.41 years; SD_age_ = 5.23 years). Findings revealed a statistically significant correlation between emotional divorce, depression, anxiety, and loneliness. One-way ANOVA confirmed that those with high levels of emotional divorce concurrently scores higher on the depression, anxiety, and loneliness tools, followed by those with moderate and low emotional divorce scores, respectively. Linear regression analysis indicated that depression, anxiety, and loneliness were strong predictors of emotional divorce, explaining 61% of the variance in the emotional divorce scores in this sample. These findings highlight the need to focus on the mental health outcomes of individuals experiencing emotional divorce, especially in societies where legal divorce may not be acceptable or encouraged. The need for regular evaluation and timely interventions for individuals struggling with mental health problems, and for restoring a healthy marital relationship is also highlighted.

## Introduction

Quality of the marital relationship determines the mental health of married individuals [1]. Therefore, mental health professionals attach special importance to the study of family and marital life [2]. Differences in attitudes, beliefs, and expectations pertaining to the spousal relationship may lead to an imbalance in the marital relationship [3]. The inability to reconcile these differences may lead to divorce.

Divorce can be classified into two types, official/legal divorce, and “emotional divorce.” The former occurs when the couple separates legally and each of them receives a divorce document [4]. Historically, formal divorce has been restricted by religion, social norms, and political influences because marriage is considered essential to the maintenance of the family system [5]. Though the acceptance of legal divorce has increased over time [6], several traditionalist societies continue to discourage legal separation of married couples. Oftentimes, owing to sociocultural norms that place special social and spiritual value onto marriage, dissatisfied couples continue to maintain cohabitation to create an illusion of continuing the marital relationship.

According to Damo and Cenci [4], emotional divorce may not necessarily precede formal/legal divorce; it may occur even before or after legal separation. While such couples endeavor to preserve the “family structure,” in reality, the family is empty inside [7]. In the absence of love and satisfaction, their married life becomes cold and distant. This dissatisfaction may have severe negative effects on the mental health of the couple [4, 8]. Furthermore, the deterioration of the marital relationship and marital compatibility lead to a loss of trust, respect, and love towards one’s spouse [9]. Instead of forming positive relationships and supporting each other, the couple behaves in a way that leads to psychological or physical harm, a sense of defeat, or low self-esteem, and both spouses attempt to find fault in the other person [10]. Couples experiencing emotional divorce often exhibit a lack of intimacy, apathy towards their spouse’s feelings, and emotional distance [7, 8, 11].

### Emotional divorce and mental health

Emotional divorce is also linked to several adverse mental health outcomes and maladaptive behavior patterns [12]. Marital satisfaction is one of the most important determinants of mental health, emotional stability, and marital success [13]. Persistent marital discord leads to impaired behavioral and emotional functioning [14], and are therefore a common reason for obtaining mental health interventions from professionals [15]. Several studies have found that marital discord is associated with subclinical depression, psychological distress [e.g., 16–18], major depressive disorders, and panic disorders [19–21]. Amato [22] reported that divorced individuals are more likely to experience depression, poor life satisfaction, and health problems. Although divorce may be a significant stressor, it is often accompanied by feelings of helplessness, depression, anxiety, aggression, sadness, guilt, and loneliness [2, 23, 24]. Overbeek et al. [25] suggested that, rather than the divorce itself, the marital conflict preceding it causes the onset of mental health problems. Thus, the repercussions of emotional divorce on the mental health of couples may be very similar to those of formal divorce.

For instance, a qualitative study on 17 married women in Jordan confirmed that while emotional divorce was most closely related to lack of intimacy and emotional distance, it also influenced women’s mental, physical, and sexual well-being [26]. Similarly, another study on married female students in a university in Jordon studied the relationship between emotional divorce and psychological hardiness, which pertains to an individual’s ability to maintain their physical and mental health when facing life’s stressors. Findings revealed that the two variables had a negative association. The authors suggested that women who exhibited high psychological hardiness were likely to possess better coping skills, which in turn acted as a protective factor against emotional divorce [27].

The links between marital dissatisfaction or discord, including divorce, and poor mental health, such as higher incidence of depression and anxiety, are well established. However, other than a handful of small studies on very specific target groups, it has not been verified if there is an association between emotional divorce and mental health outcomes. Furthermore, similar studies on Saudi women are difficult to come by. Therefore, the present study aimed to explore the relationship between emotional divorce and mental health outcomes such as anxiety, depression, and loneliness. The relevance of this study in the Saudi context is further explained in the next section.

### The Saudi context

Divorce is a complex process, and although it is not prohibited in Islam, spouses are not encouraged to separate or divorce, especially in societies with strict cultures [11]. The Saudi culture maintains a very cohesive and powerful social process structured around the family unit [28, 29]. Divorce is strongly discouraged in Saudi Arabia owing to the prevailing culture and traditions. Therefore, couples who may feel dissatisfied with their marital relationship prefer not to formally divorce. Further, the economic burden that divorce places on the family could be a strong deterrent for couples [30].

Another factor pertains to women’s status in the Saudi social system. Owing to deep-rooted and systemic patriarchy, Saudi women often face substantial economic and legal inequalities in heirship, property rights, and ownership/division of assets, which is an additional motivation for them to remain legally married despite being unhappy [31]. Additionally, Saudi Arabia’s Islamic law allows men to declare “talaq” or formal divorce, but women have to formally petition the court with clear explanations on why they cannot live with their husband anymore. Alternatively, women can obtain divorce from their husband by offering monetary compensation, an option that is not viable for most women [31, 32]. These and several other disadvantageous legal provisions render it extremely difficult for women to obtain formal divorce. Thus, a gender-biased political, legal, and social system may indirectly force several Saudi women to continue to remain in a dysfunctional marriage, simultaneously making emotional divorce inevitable.

Several couples are forced to continue to live together in the same house, separate from each other, and live in emotional isolation without any exchange of feelings [9]. Over time they may become hostile to each other, trust between them is weakened, and conflicts and disagreements arise continuously [10]. This marital discord often leads to negative psychological, social, and behavioral repercussions such as depression, anxiety, psychological loneliness, aggression, and psychosomatic disorders both in the couple and their children [4, 30, 33].

Recently, global divorce rates have risen rapidly, possibly due to economic, social, and/or cultural shifts [34, 35]. This has also been observed in countries with more stringent social norms that pose several cultural and socio-economic obstacles to the divorce process; for example, Saudi Arabia [11]. In 2020, 38.36% of marriages in Saudi Arabia ended in divorce, a 12.7% increase as compared to the rate in 2019. These percentages are higher than the global average of 18–22% [36]. These statistics only reflect the incidence of formal or legal divorce. However, emotional divorce rates are unknown, as it is difficult to determine their incidence accurately.

Studies have suggested that the incidence of emotional divorce may be twice that of formal divorce [37]. Although several studies have pointed to the repercussions of official divorce on mental health, both nationally and internationally [13, 22], those of emotional divorce on the mental health of couples in more traditionalist societies needs further research attention. Specifically, as mentioned before, Islam strongly discourages divorce, which may lead several unhappy couples to resort to staying together and remain legally married. It will be interesting to study emotional divorce in such societies.

More recently, studies on emotional divorce in Islamic countries such as Saudi Arabia, Jordan and Iran have emerged. Understandably, as this is a relative new research area, majority of these studies focus on the causes or predictors of emotional divorce, including interpersonal factors such as loss of love, respect, intimacy, and trust, poor communication, poor coping skills, poor self-efficacy, and emotional disconnect [e.g.,^38^–^42^]; and socio-cultural and economic factors such as mismatch in expectations about marriage, religious beliefs, gender roles and status, child-rearing pressures, financial pressures, and work-related pressures [e.g., 10, 12, 42–46].

Among the abovementioned studies that identified psychological factors related to emotional divorce, very few were conducted in Saudi Arabia. In the male dominant Saudi society, the cultural, religious, legal, and economic pressures on women act as a deterrent for their pursuit of legal divorce in a dysfunctional marriage. With well-established gender-based differences in emotional skills and social roles among men and women, we considered it important to study the psychological repercussions of emotional divorce on married women in Saudi Arabia. Therefore, the present study attempted to examine the relationship between emotional divorce and mental health in this population.

## Method

### Sample

We used G*Power version 3.1.9.7 to compute the recommended sample size for one-way ANOVA and linear regression. Inputting 0.05 α error probability and 80% power (and 3 independent variables for the regression), the resultant recommendation was 158 and 77 participants, respectively. Therefore, 158 was decided as the minimum required sample for this study. Purposive sampling was used to recruit participants based on the following pre-determined selection criteria: volunteering to participate in the study, being aged over 18 years, being currently married, being able to read Arabic, being citizens of Saudi Arabia, not having any intellectual disabilities, and not having a prior formal diagnosis of a mental illness. Of the 282 women who expressed interest in the study, 8.86% withdrew consent during data collection and 5.67% were excluded due to incomplete data. The final sample comprised 241 married Saudi women, with a mean age of 34.41 years (SD = 5.23 years). Thus, despite losing about 14% of the sample, this study met the minimum requirement of 158 participants substantially.

### Measures

#### Emotional divorce scale

The Arabic 37-item Emotional Divorce Scale (EDS) developed by [47]. was used to assess the level of emotional divorce in the sample. Its items are structured across three dimensions: social (11 items), psychological (13 items), and emotional (13 items). Each item is rated on a 5-point Likert scale (1 = never, 2 = rarely, 3 = sometimes, 4 = always, and 5 = always), with total scores ranging from 37–185 points. The degree of emotional divorce is classified into three levels; high, medium, and low, using the following equation:

$$\frac{highest\;response-lowest\;response}{Number\;of\;categories}=\frac{5-1}{3}=\frac{4}{3}=1.33$$

Accordingly, scale scores are classified as follows: < 2.33 as low emotional divorce, 2.34–3.67 as moderate emotional divorce, and >3.68 as high emotional divorce.

The scale was validated by examining its internal consistency through interrelationships between elements, with scores ranging from 0.58–0.67. The Cronbach alpha coefficient for each dimension ranged from 0.77–0.86, and that for the total score was 0.79. The split-half coefficient was 0.89. Thus, the EDS was thus determined to possess high validity [47].

#### Centre for epidemiological studies depression scale (CES-D-10)

The CES-D-10 scale consists of ten items to measure the level of depression symptoms during the week preceding the assessment [48]. It contains three items on depressive affect, five on physical symptoms, and two on positive affect. Of the ten items, eight evaluate positive symptoms, while two (Item 5 and 8) evaluate the negative symptoms of depression. Each items is rated on a four-point Likert scale ranging from "rarely or none of the time" (0) to "all the time" (3). Item 5 and 8 are reverse-scored as they are positively formulated. Overall scores range from 0 to 30, with higher scores indicating greater severity of depressive symptoms. In the original study, the CES-D-10 exhibited good internal consistency and reliability [48]. In the current study, it had high internal consistency (Cronbach α = .85).

#### Generalized anxiety disorder questionnaire (GAD-7)

The GAD-7 scale was developed to identify possible causes of generalized anxiety disorder and to measure the severity of symptoms [49]. The questionnaire consists of seven items that ask respondents how often they have felt distressed in the past two weeks, with each item representing seven main symptoms of generalized anxiety disorder. Answer options range from 0 to 3 ("not at all" to "almost every day"). The total score ranges from 0 to 21, with scores of 5, 10, and 15 representing mild, moderate, and severe anxiety symptoms, respectively. The scale showed excellent internal consistency (Cronbach α = 0.92) and reliability (correlation within the class = .83) in the original study [49]. Subsequently, several studies have shown high internal consistency, test-retest reliability, convergence, construction, standards, and validation [e.g., 50, 51].

#### The De Jong Gierveld 6-item loneliness scale (DJGLS)

The DJGLS [52] uses three items each, to assess the level of social (e.g., "there are many people I can completely trust") and emotional (e.g., "I feel a general sense of emptiness") loneliness experienced by individuals in their interpersonal relationships. Each items is rated on a 5-point Likert scale ranging from “strongly disagree” to “strongly agree.” The tool exhibited a Cronbach’s alpha (α) of 0.76 in the original study [52]. Subsequently, several studies have proven the reliability of this scale [52, 53]. In the current study, it exhibited good internal consistency (Cronbach α = .76).

For use in the present study, the GAD-7, CES-D-10, and DJGLS were translated from English to Arabic. First, the English version was translated into Arabic by a bilingual professor from the Department of English at the Faculty of Languages and Translation at the researcher’s organization. The Arabic version was then retranslated into English by another professor who specializes in English and whose first language is Arabic. The Arabic and English translated versions of the scale were then reviewed by three specialists in Arabic, Psychology, and English, respectively. Based on the consensus among the three specialists, some words and items were revised to create the final Arabic version of the study tools.

### Data collection procedure

Data were collected in September 2022. Before collecting the data, the researchers obtained ethics approval from the Deanship of Scientific Research at Princess Nourah bint Abdulrahman University, Saudi Arabia (FRP-1443-25). All procedures performed were in accordance with the ethical standards of the institutional research committee and with the 1964 Declaration of Helsinki and its later amendments or comparable ethical standards. Potential participants were informed of the possibility of volunteering if they wished to participate in this study via social media. They were provided a web link that led to information about the study’s purpose and procedures. All potential participants were informed about their right to voluntary participation, confidentiality, and freedom to withdraw from the study. Only those who voluntarily provided their consent on the web form continued to complete the study tools. Data collection was completed in about two months.

### Data analysis

All statistical analyses were performed using SPSS version 20. Descriptive statistics included frequency, percentage, mean, and standard deviation, as applicable. To study the relationship between emotional divorce and the mental health components, one-way ANOVA, correlation coefficients, and linear regression analysis was used.

## Results and discussion

### Emotional divorce

The mean score on the EDS was 117.47 (SD = 21.002; theoretical range: 37–185). Using the tool’s classification of low, moderate, and high scores, it was found that majority of the participants (about 78%) experienced moderate-severe levels of emotional divorce (Table 1).

**Table 1:** Prevalence of emotional divorce among Saudi married women.

Emotional divorce levels	Emotional divorce scaled scores	Frequency (N = 241)	Percentage
**Low**	≤ 2.33	52	21.58%
**Moderate**	2.34–3.67	148	61.41%
**High**	≥ 3.68	41	17.01%

Emotional divorce signifies the presence of marital discord. As explained in the Introduction section, several couples end up living with emotional divorce primarily due to socio-cultural factors such as a negative attitude towards formal divorce, fear of losing face in the society, lack of decision-making ability, fear of an uncertain future, and fear of loneliness and social ostracization. Such couples maintain cohabitation even in the absence of an emotional and sexual connection with their spouse [9]. Thus they experience emotional divorce and loneliness but do not talk about obtaining formal divorce [54]. Therefore, despite increasing acceptance and incidences of formal divorce globally (18–22%) and in Saudi Arabia (38.38% in 2020) [36], researchers opined that the incidence of emotional divorce would be higher. In fact, Afrasaibi [37] suggested that the incidence of emotional divorce could be double that of formal divorce. The present finding of 78% incidence of moderate to high levels of emotional divorce corroborates this opinion as well as findings of similar studies conducted in countries such as Iran, Jordan, and Palestine [7, 27, 55–57] Furthermore, considering the patriarchal social structure of Saudi Arabia that affords a weaker social, economic, and legal status for women; the traditionalist religious and cultural beliefs that consider the marital relationship divine, and therefore, discourage divorce; and the social stigma associated with divorce, the high emotional divorce levels among the present sample are understandable.

### Relationship between the study variables

To study the relationship between emotional divorce and mental health components, first, a one-way ANOVA was used to examine if depression, anxiety, and loneliness scores differed across the three levels of emotional divorce. Findings revealed significant group differences in all three mental health outcomes (Table 2).

**Table 2:** Results of the one-way ANOVA comparing between-group differences in mental health outcomes across emotional divorce levels.

Mental Health Outcomes	Sum of Squares	df	Mean Square	F	p
**Depression**	620.97	2	310.48	33.541	.000
**Anxiety**	332.54	2	166.27	36.016	.000
**Loneliness**	713.47	2	356.74	60.427	.000

Group-wise descriptive statistics for the 3 mental health outcomes have been presented in Table 3.

**Table 3:** Descriptive statistics for mental health outcomes across Emotional divorce levels.

Emotional divorce levels	Depression M (SD)	Anxiety M (SD)	Loneliness M (SD)
**Low**	18.73 (3.24)	14.17 (2.53)	18.63 (2.73)
**Moderate**	21.8 (3.01)	16.68 (2.08)	21.37 (2.55)
**High**	23.76 (2.9)	17.66 (1.87)	24.2 (1.35)

*Note*. The theoretical range of scores for depression was 0–30, for anxiety was 0–21, and for loneliness was 0–30.

Post-hoc analyses using Scheffe’s test revealed that those with high emotional divorce levels had the highest mean scores on depression, anxiety, and loneliness, followed by the moderate and low emotional divorce groups, respectively (p < 0.05 for all comparisons). Together, findings of the one-way ANOVA and post-hoc tests suggest that individuals with higher levels of emotional divorce were also likely to exhibit higher scores on depression, anxiety, and loneliness. Furthermore, a correlation analysis revealed that all three mental health outcomes had a moderate to strong positive correlation with emotional divorce (Table 4). Again, this finding suggests that an increase in emotional divorce scores led to a consequent increase in depression, anxiety, and loneliness scores, and vice-versa.

**Table 4:** Correlation between Emotional divorce and mental health components.

Study variables	Emotional divorce	Depression	Anxiety	Loneliness
**Emotional divorce**	-	0.64	0.55	0.72
**Depression**	0.64	-	0.57	0.68
**Anxiety**	0.55	0.57	-	0.64
**Loneliness**	0.72	0.68	0.64	-

*Note*. For all correlations p < 0.01 (2-tailed).

However, it is important to note that, as the present study utilized a cross-sectional design with a limited sample, the directionality of this correlation cannot determine causality. That is, based on the present data, we cannot determine whether emotional divorce scores caused an increase in depression, anxiety, and loneliness scores or whether the mental health outcomes caused an increase in emotional divorce.

To further establish the nature of association between the study variables, we conducted linear regression analysis (Table 5) with emotional divorce as the dependent variable, and depression, anxiety, and loneliness as independent variables.

**Table 5:** Regression analysis results examining the association of Emotional divorce (dependent variable) with depression, anxiety, and loneliness (independent variables).

Variables	B	SE	Beta	t	p
**Constant**	28.48	4.75		5.99	0.001
**Depression**	0.99	0.247	0.224	4.04	0.001
**Anxiety**	1.76	0.309	0.288	5.71	0.001
**Loneliness**	2.05	0.308	0.40	6.68	0.001

*Note*. R = 0. 788; R^2^ = 0.62; Adjusted R^2^ = 0.61; F (131.54); p < .01.

As evident from Table 5, the three independent variables together explained 61% of the variance in the emotional divorce score. Further, findings revealed that a 1-point increase in depression scores led to a nearly similar increase in the emotional divorce score. Similarly, a 1-point increase in anxiety scores led to a 1.76-point increase in the emotional divorce score. Finally, a 1-point increase in loneliness scores led to a 2-point increase in the emotional divorce score, which was supported by the strongest correlation coefficient for these two variables (Table 4).

Mental health problems such as depression, anxiety, and loneliness are common among individuals experiencing formal and emotional divorce alike. Therefore, it seems reasonable to assume that emotional divorce in poor marital relationships could be predicted based on the incidence of mental health problems in the present sample. The results of the one-way ANOVA, correlation coefficient, and linear regression threw light on the nature of association between emotional divorce and measures of adverse mental health outcomes. was confirmed by studies that reported that emotional divorce leads to depression, impatience, anxiety, psychological loneliness, feelings of inferiority, and loss of self-confidence [42, 58]. Thus, the quality of marital relationships has a strong influence on overall physical and mental well-being. Specifically, a poor marital relationship could lead to depressive symptoms and anxiety [59]. Several studies have confirmed that both men and women with mental health problems also reported higher rates of marital problems [60–62]. Interpersonal theories of mental illness [63, 64] purport that mental illness in one spouse may have negative effects on the patterns of interaction and communication between both spouses, thus disrupting routines, generating pressure and burden on the relationship, provoking marital disputes, and reducing the quality of the marital relationship. Whisman [65] reported that a husband or wife’s depression was independently associated with marital quality ratings or good communication patterns. Thus, the interpersonal aspects of mental disorders at the individual level may influence the dyadic relationship among spouses, the quality of their relationship, and, in doing so, it may increase the risk of marital relationship discord [66]. Another study reported an association between how couples manage their differences in a marriage and their mental health status [67]. Similarly, a study by Halford et al. [68] on the association between marital problems and symptoms of depression, anxiety disorders, loneliness, alcohol use, and functional psychosis, found that that marital problems often aggravated the mental health issues.

The present study focused on women owing to the added social, emotional, financial, and legal deterrents they experience in seeking formal divorce in Saudi Arabia. Therefore, Saudi married women may be less likely to pursue divorce proceedings than would men. However, as evident from the present results, emotional divorce is strongly associated with adverse mental health outcomes among Saudi women. Though it would be interesting to examine if married men in Saudi Arabia show similar findings, unfortunately, that was beyond the purview of the present study. Nevertheless, some aspects of the way women handle marital discord may help further explain the present results.

Du Rocher Schudlich et al. [59] suggested that women who are dissatisfied with their marriage may avoid discussing their feelings; specifically, to avoid conflict with the husband. However, despite avoiding conflict in the short term, such avoidance could lead to low self-esteem and high self-denial, in turn increasing their susceptibility to long-term symptoms of depression and anxiety. Relatedly, a longitudinal study by Fincham et al. [69] found that, for women, marital relationship disorder or marital dissatisfaction was a major cause of depression. Another study confirmed that, for married women, high levels of marital discord predicted a subsequent rise in depressive symptoms over time [70].

Similarly, Proulx et al. [71] found that marital hostility to husbands was significantly associated with increased depressive symptoms in women. Studies have consistently shown that mental disorders are often a precursor and possibly a reason for the dissolution of a marriage. However, mental disorder may be caused by the conflict and dissatisfaction with the relationship that exists long before formal divorce is obtained [72]. Furthermore, unhappiness in marriage can lead to increased emotional distress in marital interactions, which may lead to emotional divorce. This, in turn, would increase women’s vulnerability to depression, anxiety, and loneliness in multiple contexts.

### Limitations of the study

While this study offers interesting insights on Saudi women’s experience of emotional divorce and its association with depression, anxiety, and loneliness, it has several limitations that need to be considered when attempting to generalize these findings to other contexts. First, the present sample was biased owing to the sampling and data collection methods chosen. As we contacted volunteers through social media, it only included women with access to social media, internet services, and possibly education too. These participants may not be representative of the general population of women in Saudi Arabia. Furthermore, the sample is not divergent enough to reflect the social reality of Saudi Arabia, which includes women who do not meet one or more of the selection criteria set for this study. However, an online data collection method and the related sampling method was the only way to provide potential participants a sense of anonymity, which was necessary for them to open up about marital discord, a fact several women in a traditional society like Saudi Arabia may not be willing to accept.

Further, the sample size was quite small to be nationally representative, but it was estimated using the G*Power tool’s recommendations for preset power and error limits. Therefore, the sample of 241 women offers some reliability to the present analyses. Nevertheless, it is recommended that future studies include a larger, more diverse and representative sample to provide a clearer picture of the general population. Finally, future comparative studies including males and females would provide a more holistic understanding of the core variables studied.

This study could also have been improved by considering the other factors influencing any or all of the present variables. It is strongly recommended for future studies to simultaneously study other individual, family, social, cultural, religious, and economic factors. Finally, the cross-sectional nature of this study did not enable us to establish causality among the studied variables. Indeed, emotional divorce and mental health problems could have a bidirectional relationship.

Future studies should attempt to overcome the present limitations to derive more holistic and robust insights. Nevertheless, the present study holds merit in that it is one of the very few studies focusing on this topic in this specific target population.

## Conclusions and implications

The present study revealed a positive correlation between depression, anxiety, loneliness, and emotional divorce. Additionally, mental health issues predicted 61% of the variance in emotional divorce. Further, the depression, anxiety, and loneliness experienced by married women in Saudi Arabia affected the level of emotional divorce they experienced, and vice versa. This study provides strong evidence for the negative impact of emotional divorce on the mental health of married women. Nevertheless, it is as likely to affect their husbands and children as well. Therefore, it is important to recognize this as a public health concern and to develop appropriate strategies to control emotional divorce levels in the Saudi society.

In particular, the need to educate those who are about to get married about the nature of married life, ways to deal with their life partner, and healthy coping strategies for dealing with marital problems is clear. Additionally, at the individual and social level, it is important to facilitate and encourage emotional and romantic expression with one’s life partner because it would strengthen the marital relationship, increase changes of success, and reduce the possibility of emotional divorce. In turn, mental health problems such as depression, anxiety, and loneliness could be prevented. Finally, regular assessment of individual mental health and the quality of the marital relationship could help identify mental health and marital problems before they become serious issues. Therapy for couples, especially behavioral couples therapy (BCT), is an important component of effective treatment for depression, anxiety disorders, and loneliness within the marital context.

## Supporting information

S1 File (DOCX)

S2 File (DOCX)
