# Supplementary material for: Impact of emotional divorce on the mental health of married women in Saudi Arabia
Source: PLoS One. 2023 Nov 10;18(11):e0293285. doi: 10.1371/journal.pone.0293285 (PMC10637691; doi:10.1371/journal.pone.0293285)
Supplement: S1 File — (DOCX) [file pone.0293285.s001.docx]

Emotional Divorce Questionnaire

<https://docs.google.com/document/d/1OJNSVsqM3mOG2lp_7TsDaWIwUJ1jYoxXqqKkI1iep0M/edit?usp=sharing>

Table 1. Prevalence of Emotional Divorce among Saudi Married Women.

<https://docs.google.com/document/d/18ZLtiCpfNONK7C7LyVc8w5BSvq8cQeuuN_9zIL3j9bU/edit?usp=sharing>

Table 2. Results of the One-way ANOVA Comparing Between-Group Differences in Mental Health Outcomes across Emotional Divorce Levels.

<https://docs.google.com/document/d/13oaCJAjdvQFeDtE78FyCl4-XMRW86l9opVlk8fBSL5s/edit?usp=sharing>

Table 3. Descriptive Statistics for Mental Health Outcomes across Emotional Divorce Levels.

<https://docs.google.com/document/d/1OmKWnwgPUupx4erQpwIRL6rAFmLKJNCF3bxvU_6W3Lc/edit?usp=sharing>

Table 4. Correlation between Emotional Divorce and Mental Health Components.

<https://docs.google.com/document/d/1Fj_lkqQY98YdnDJLIXH-vWAgnf2EuHMUIao86Jan9E4/edit?usp=sharing>

Table 5. Regression Analysis Results Examining the Association of Emotional Divorce (dependent variable) with Depression, Anxiety, and Loneliness (independent variables).

<https://docs.google.com/document/d/1QTKSLmLO9g8vJ8ZJaUOymqViK7pDr8GGO5DFpeSZc0g/edit?usp=sharing>
