# Supplementary material for: Impact of emotional divorce on the mental health of married women in Saudi Arabia
Source: PLoS One. 2023 Nov 10;18(11):e0293285. doi: 10.1371/journal.pone.0293285 (PMC10637691; doi:10.1371/journal.pone.0293285)
Supplement: S2 File — (DOCX) [file pone.0293285.s002.docx]

**Emotional Divorce Questionnaire**

Al-Shahrani, H. F., & Hammad, M. A. (2023). Impact of emotional divorce on the mental health of married women in Saudi Arabia. *PLOS ONE* .‏

|  | Questionnaire items | Always | Often | Sometimes | Rarely | Never |
| --- | --- | --- | --- | --- | --- | --- |
| 1 | I feel not having fun when going out with my husband at social events. |  |  |  |  |  |
| 2 | Lack of material income affects my emotional relationship with my husband. |  |  |  |  |  |
| 3 | My husband blames me a lot if I misbehave with money. |  |  |  |  |  |
| 4 | I feel that my husband's relationship with my parents and relatives is bad. |  |  |  |  |  |
| 5 | I save money without my husband's knowledge. |  |  |  |  |  |
| 6 | I forget about my family when I leave home for several days. |  |  |  |  |  |
| 7 | When my parents come to visit us, my husband does not take the initiative to receive them. |  |  |  |  |  |
| 8 | I feel compelled to continue the marital relationship just for the sake of my children. |  |  |  |  |  |
| 9 | My husband shows my negative personality aspects in front of others. |  |  |  |  |  |
| 10 | My husband compares me to others and feels inferior compared to them. |  |  |  |  |  |
| 11 | I don't trust my husband. |  |  |  |  |  |
| 12 | When I get angry, I humiliate my husband. |  |  |  |  |  |
| 13 | When I feel anxious, stressed, my husband does not care about me. |  |  |  |  |  |
| 14 | My husband is not interested in me in most situations. |  |  |  |  |  |
| 15 | On free days, holidays I sit in a single room away from my husband. |  |  |  |  |  |
| 16 | I get bored with my husband all the time |  |  |  |  |  |
| 17 | When we face a problem, we don't try to solve it together. |  |  |  |  |  |
| 18 | I'm having the thought of divorcing my husband. |  |  |  |  |  |
| 19 | I feel that my husband does not share my worries. |  |  |  |  |  |
| 20 | I ignore my husband's news. |  |  |  |  |  |
| 21 | I don't share my husband's work worries. |  |  |  |  |  |
| 22 | I don't talk to my husband about anything about me. |  |  |  |  |  |
| 23 | When returning from work we do not care for each other. |  |  |  |  |  |
| 24 | My husband does not sympathize with me when I am under strong pressure. |  |  |  |  |  |
| 25 | My husband is fit to be a father, not a husband. |  |  |  |  |  |
| 26 | I feel that my feelings are cold towards me, my husband. |  |  |  |  |  |
| 27 | My husband and I don't exchange congratulations on occasions. |  |  |  |  |  |
| 28 | My husband ignores me after doing a good job that cost me a lot of effort. |  |  |  |  |  |
| 29 | I feel unhappy about my married life. |  |  |  |  |  |
| 30 | I get bored when I hear my husband talking. |  |  |  |  |  |
| 31 | Anna is very unhappy with my marriage. |  |  |  |  |  |
| 32 | I feel that my husband is unable to make me happy. |  |  |  |  |  |
| 33 | I feel that the relationship between me and my husband is a sign of separation. |  |  |  |  |  |
| 34 | Our sexual relationship is almost non-existent. |  |  |  |  |  |
| 35 | I get upset when my husband approaches me. |  |  |  |  |  |
| 36 | I feel that my husband is not jealous of me as he should. |  |  |  |  |  |
| 37 | I'm disappointed with my husband. |  |  |  |  |  |
